# A Learning Framework of Nonparallel Hyperplanes Classifier

**DOI:** 10.1155/2015/497617

**Published:** 2015-06-16

**Authors:** Zhi-Xia Yang, Yuan-Hai Shao, Yao-Lin Jiang

**Affiliations:** ^1^College of Mathematics and Systems Science, Xinjiang University, Urumqi 830046, China; ^2^State Key Lab of Biochemical Engineering, Institute of Process Engineering, Chinese Academy of Sciences, Beijing 100190, China; ^3^Zhijiang College, Zhejiang University of Technology, Hangzhou 310024, China

## Abstract

A novel learning framework of nonparallel hyperplanes support vector machines (NPSVMs) is proposed for binary classification and multiclass classification. This framework not only includes twin SVM (TWSVM) and its many deformation versions but also extends them into multiclass classification problem when different parameters or loss functions are chosen. Concretely, we discuss the linear and nonlinear cases of the framework, in which we select the hinge loss function as example. Moreover, we also give the primal problems of several extension versions of TWSVM's deformation versions. It is worth mentioning that, in the decision function, the Euclidean distance is replaced by the absolute value |*w* ^T^ *x* + *b*|, which keeps the consistency between the decision function and the optimization problem and reduces the computational cost particularly when the kernel function is introduced. The numerical experiments on several artificial and benchmark datasets indicate that our framework is not only fast but also shows good generalization.

## 1. Introduction

Classification problem is an important issue in machine learning and data mining, which is mainly comprised of binary and multiclass classification. Support vector machine (SVM), proposed by Burges [1] and Cortes and Vapnik [2], is an excellent tool for classification. In contrast with conventional artificial neural networks (ANNS) which aim at reducing empirical risk, SVM is principled and implements the structural risk minimization (SRM) that minimizes the upper bound of the generalization error [3–5]. Within a few years after its introduction, SVM has been successfully applied to pattern classification and regression estimation like face detection [6, 7], text categorization [8], time series prediction [9], bioinformatics [10], and so forth.

Recently, for binary classification, Mangasarian and Wild [11] proposed the generalized eigenvalue proximal support vector machine (GEPSVM) via two nonparallel hyperplanes. In their approach, the data points of each class are proximal to one of two nonparallel hyperplanes. The nonparallel hyperplanes are determined by eigenvectors corresponding to the smallest eigenvalues of two related generalized eigenvalue problems. Inspired by GEPSVM [11], Jayadeva et al. [12] developed twin SVM (TWSVM) with two nonparallel hyperplanes. However, the two hyperplanes are got by solving two quadratic programming (QP) problems, similar to the standard SVM. Furthermore, TWSVM differs from the standard SVM in fundamental way. In TWSVM, one solves a pair of smaller size QP problems rather than a single QP problem in the standard SVM. Therefore, TWSVM works faster than the standard SVM. Subsequently, there are many extensions for TWSVM including the improvements on TWSVM (TBSVM) [13], the least square TWSVM (LS-TWSVM) [14–17], nonparallel plane proximal classifier (NPPC) [18], smooth TWSVM [19], geometric algorithm [20], and twin support vector regression (TWSVR) [21]. TWSVM was also extended to deal with multiclassification TWSVM [22–24]. More precisely, in [22], TWSVM was extended straight from binary classification to multiclass classification, in which each primal problem covers all patterns except the patterns of the *k*th class in the constraints for the *k*th (*k* = 1,2,…, *K*) hyperplane. In [23], the authors extended TWSVM based on the idea of “one-versus-rest” (1-v-r) from binary classification to multiclass classification, in which there are two quadratic programming (QP) problems for each reconstructing binary classification. However, they both have not kept the advantage of TWSVM which has lower computational complexity than that of the standard SVM. In [24], Yang et al. proposed multiple birth SVM (MBSVM) with much lower computational complexity than that of both [22, 23] by solving *K* smaller size of QP problems for *K*-class classification; only the empirical risk is considered like TWSVM. However, in TBSVM [13], the structural risk minimization principle is implemented by introducing the regularization term.

In this paper, we propose a novel learning framework of nonparallel hyperplanes support vector machines based on TWSVM and its extension versions, called NPSVMs, which not only provide a unified view for TWSVM and its many extension versions but also can deal with binary and multiclass classification problems. For binary classification, if the loss function is the hinge loss function, then the framework can become TWSVM [12] or TBSVM [13] with different parameters; if the loss function is the square loss function, then the framework is LS-TWSVM [14]; if the loss function is the convex combination of the linear and square loss functions, then the framework is NPPC [18]. Actually, we can also get smooth TWSVM [19] by replacing 2-norm with 1-norm in the framework. However, for multiclass classification, the framework does not directly extend, in which we switch the roles of the patterns of the *k*-th class and the rest class and replace “min” with “max” in the decision function. Moreover, we only use the absolute value |*w* ^T^ *x* + *b*| rather than the Euclidean distance in the decision function due to the twofold reasons: reducing the computational cost particularly when the kernel function is introduced and making the consistency since it is the corresponding absolute value that appears in the primal problems. Concretely, we discuss the linear and nonlinear cases of the framework, in which we select the hinge loss function as example. Moreover, we also give the primal problems of extensions of LS-TWSVM, 1-norm LS-TWSVM, NPPC, and smooth TWSVM. Finally, the numerical experiments on several artificial and benchmark datasets indicate that our frameworks are not only fast but also show good generalization.

The paper is organized as follows.  Section 2 introduces the brief reviews of SVMs.  Section 3 proposes our frameworks, in which Section 3.1 discusses the linear framework, Section 3.2 extend into the nonlinear framework, Section 3.3 gives SOR algorithm for solving the hinge NPSVMs, and Section 3.4 discusses several other extension approaches. Finally, Section 4 deals with experimental results and Section 5 contains concluding remarks.

## 2. Brief Reviews of SVMs

### 2.1. Twin Support Vector Machine

Given the following training set for the binary classification:

(1) $$\begin{array}{c} \;\begin{array}{c} T=\left\{\left(x_{1},y_{1}\right),\ldots ,\left(x_{l},y_{l}\right)\right\}, \end{array} \end{array}$$

where (*x*_*i*_, *y*_*i*_) is the *i*th data point, the input *x*_*i*_ ∈ *R* ^*n*^ is a pattern, the output *y*_*i*_ ∈ {1,2} is a class label, *i* = 1,…, *l*, and *l* is the number of data points. In addition, let *l*_1_ and *l*_2_ be the number of data points in positive class and negative class, respectively, and *l* = *l*_1_ + *l*_2_. Furthermore, the matrices *A*_1_ ∈ *R* ^*l*_1_×*n*^ and *A*_2_ ∈ *R* ^*l*_2_×*n*^ consist of the *l*_1_ inputs of Class 1 and the *l*_2_ inputs of Class 2, respectively.

The gaol of TWSVM [12] is to find two nonparallel hyperplanes in *n*-dimensional input space:

(2) $$\begin{array}{c} x^{T}w_{1}+b_{1}=0, \end{array}$$

(3) $$\begin{array}{c} x^{T}w_{2}+b_{2}=0, \end{array}$$

such that one hyperplane is close to the patterns of one class and far away from the patterns of the other class to some extent. TWSVM is in spirit of GEPSVM [11]. But both of GEPSVM and TWSVM are different from the standard SVM. For TWSVM, each hyperplane is generated by solving a QP problem looking like the primal problem of the standard SVM. The primal problems of TWSVM can be presented as follows:

(4) minw1,b1,ξ2 12A1w1+e1b122+C1e2Tξ2,s.t. H−A2w1+e2b1+ξ2≥e2,HHHHξ2≥0;

(5) minw2,b2,ξ1 12A2w2+e2b222+C2e1Tξ1,s.t.  A1w2+e1b2+ξ1≥e1,HHHHξ1≥0,

where *C*_1_ and *C*_2_ are nonnegative parameters and *e*_1_ and *e*_2_ are vectors of ones of appropriate dimensions. In the QP problem (4), the objective function tends to keep hyperplane (2) close to the patterns of Class 1 and the constraints require the hyperplane (2) to be at a distance of at least 1 from the patterns of Class 2. The QP problem (5) has similar property. Moreover, we note that the constraints do not contain all patterns in the training set (1) but are determined by only the patterns of one class in both classes. Therefore, in [12], the authors claimed that TWSVM is approximately four times faster than the standard SVM.

Define $G=\left[\begin{array}{cc} A_{2} & e_{2} \end{array}\right]$ and $H=\left[\begin{array}{cc} A_{1} & e_{1} \end{array}\right]$. It has been shown that when both *G* ^T^ *G* and *H* ^T^ *H* are positive definites, the Wolfe duals of (4) and (5) are written as follows:

(6) maxα2 e2Tα2−12α2TGHTH−1GTα2,s.t. h0≤α2≤C1,

(7) maxα1 e1Tα1−12α1THGTG−1HTα1,s.t. h0≤α1≤C2,

respectively, where *α*_2_ and *α*_1_ are Lagrangian multipliers.

In order to avoid the possible ill-conditioning of *H* ^T^ *H* and *G* ^T^ *G*, TWSVM introduces a term *ϵI* (*ϵ* > 0), where *I* is an identity matrix of appropriate dimensions. Thus, the nonparallel hyperplanes (2) and (3) can be obtained from the solutions *α*_1_ and *α*_2_ of the QP problems (6) and (7). Consider

(8) $$\begin{array}{c} z_{1}=-{\left(H^{T}H+\epsilon I\right)}^{-1}G^{T}\alpha_{2},\;\;z_{2}={\left(G^{T}G+\epsilon I\right)}^{-1}H^{T}\alpha_{1}, \end{array}$$

where $z_{k}={\left[\begin{array}{cc} w_{k}^{T} & b_{k} \end{array}\right]}^{T}$, *k* = 1,2. Moreover, a new pattern *x* ∈ *R* ^*n*^ is assigned to Class *k* (*k* = 1,2), depending on which of the two nonparallel hyperplanes given by (2) and (3) lies closer to; that is,

(9) $$\begin{array}{c} f\left(x\right)=arg\underset{k=1,2}{min}\frac{\left|w_{k}^{T}x+b_{k}\right|}{{\left\|w_{k}\right\|}_{2}}. \end{array}$$

### 2.2. Multiple Birth Support Vector Machine

Given the training set

(10) $$\begin{array}{c} T=\left\{\left(x_{1},y_{1}\right),\ldots ,\left(x_{l},y_{l}\right)\right\}, \end{array}$$

where the input *x*_*i*_ ∈ *R* ^*n*^, *i* = 1,…, *l*, is the pattern and the output *y*_*i*_ ∈ {1,…, *K*} is the class label. The task is to seek *K* hyperplanes,

(11) $$\begin{array}{c} w_{k}^{T}x+b_{k}=0,\;k=1,\ldots ,K, \end{array}$$

and assign the class label according to which hyperplane a new pattern is farthest from.

For convenience, denote the number of data points of the *k*th class in the training set (10) as *l*_*k*_ and define the following matrixes: the patterns belonging to the *k*th class are represented by the matrix *A*_*k*_ ∈ *R* ^*l*_*k*_×*n*^, *k* = 1,…, *K*. In addition, define the matrix

(12) $$\begin{array}{c} B_{k}={\left[A_{1}^{T},\ldots ,A_{k-1}^{T},A_{k+1}^{T},\ldots ,A_{K}^{T}\right]}^{T}; \end{array}$$

that is, *B*_*k*_ ∈ *R* ^(*l*−*l*_*k*_)×*n*^ consists of the patterns belonging to all classes except the *k*th class, *k* = 1,…, *K*. The primal problems of MPSVM [24] are comprised of the following *K* QP problem:

(13) minwk,bk,ξk 12Bkwk+ek1bk2+Ckek2Tξk,s.t. hhAkwk+ek2bk+ξk≥ek2, ξk≥0,

where *e*_*k*1_ ∈ *R* ^(*l*−*l*_*k*_)^ and *e*_*k*2_ ∈ *R* ^*l*_*k*_^ are the vectors of ones, *ξ*_*k*_ is the slack variable, and *C*_*k*_ > 0 is the penalty parameter, *k* = 1,…, *K*. The dual problem of QP problem (13) is formulated as follows:

(14) maxαk ek2Tαk−12αkGkHkTHk−1GkTαk,s.t. h0≤αk≤Ck,

where the penalty parameter *C*_*k*_ > 0, $H_{k}=\left[\begin{array}{cc} B_{k} & e_{k1} \end{array}\right]$, and $G_{k}=\left[\begin{array}{cc} A_{k} & e_{k2} \end{array}\right]$, *k* = 1,2,…, *K*. Similarly, in order to avoid the possibility of the ill-conditioning of the matrix *H*_*k*_^T^ *H*_*k*_ in some situations, one introduces a regularization term *ϵI*, where *ϵ* > 0 is a fixed small scalar and *I* is the identity matrix with appropriate size.

After getting the solution ${\left[\begin{array}{cc} w_{k}^{T} & b_{k} \end{array}\right]}^{T}=-{\left(H_{k}^{T}H_{k}+\epsilon I\right)}^{-1}G_{k}^{T}\alpha_{k}$ to the above QP problem (13) with *k* = 1,…, *K*, a new pattern *x* ∈ *R* ^*n*^ is assigned to class *k* (*k* ∈ {1,…, *K*}), depending on which of the *K* hyperplanes given by (11) lies farthest from; that is, the decision function is represented as

(15) $$\begin{array}{c} f\left(x\right)=arg\underset{k=1,\ldots ,K}{max}\frac{\left|w_{k}^{T}x+b_{k}\right|}{{\left\|w_{k}\right\|}_{2}}, \end{array}$$

where |·| is the absolute value.

## 3. The Framework of Nonparallel Hyperplanes Classifiers

In this section, we propose a learning framework of nonparallel hyperplanes classifier, which gives a unified form for TWSVM and its many extension versions and extend them into multiclass classification problem. We first develop the linear framework and then extend it to nonlinear framework.

### 3.1. Linear Framework

Given the training set (10), the task is to find *K* nonparallel hyperplanes:

(16) $$\begin{array}{c} w_{k}^{T}x+b_{k}=0,\;\;k=1,2,\ldots ,K, \end{array}$$

one for each class. For obtaining the *K* unknown hyperplanes, we construct the following standard framework for each unknown hyperplane:

(17) minwk,bk12Bkwk+ek2bk22+12Ck∗wk22+bk2 +Ckek1TLek1,Akwk+ek1bk,

where the matrix *A*_*k*_ is comprised of the patterns in the *k*th class, the matrix *B*_*k*_ is defined (12), *C*_*k*_^*∗*^ ≥ 0 and *C*_*k*_ > 0 are the parameters, *e*_*k*1_ and *e*_*k*2_ are vectors of ones of appropriate dimensions, *k* = 1,2,…, *K*, and *L*(·, ·) is the loss function (e.g., square loss, hinge loss, etc.). In the optimization problem (17), the first term approximatively minimizes the sum of the squared Euclidean distances from the patterns except for the *k*th class to hyperplanes; the second term is the Tikhonov regularization term [25] and can implement the structural risk minimization principle like TBSVM [13]; the third term constitutes the loss function which is defined different loss functions corresponding to different models.

For a new pattern *x* ∈ *R* ^*n*^, we assign to class *k* (*k* = 1,2,…, *K*) according to the following decision function:

(18) $$\begin{array}{c} f\left(x\right)=arg\underset{k=1,2,\ldots ,K}{max}\left|w_{k}^{T}x+b_{k}\right|, \end{array}$$

where |·| is the absolute value. Note that we only use the absolute value |*w* ^T^ *x* + *b*| in the decision function. There are two main reasons: one is that the first term of the optimization problem (17) just minimizes the sum of the square rather than the sum of square Euclidean distance from the patterns to hyperplanes, so it should keep consistency between the optimization problem and the decision function; another is that it reduces the computational cost particularly when the kernel function is introduced afterwards.

In fact, if *K* = 2, the parameter *C*_*k*_^*∗*^ is equal to 0, and the loss function is hinge loss function, that is, *L*(1, *g*(*x*)) = max(0,1 − *g*(*x*)), then the optimization problem (17) becomes TWSVM [12]. Moreover, if the parameter *C*_*k*_^*∗*^ > 0 is alterable, then it is TBSVM [13]. And if the loss function is the square loss function, that is, *L*(1, *g*(*x*)) = (1 − *g*(*x*))^2^, it is LS-TWSVM [14]. And if the loss function is a convex combination of linear and square loss, that is, *L*(1, *g*(*x*)) = *δ*(1 − *g*(*x*))+(1 − *δ*)(1 − *g*(*x*))^2^, where *δ* ∈ (0,1), then it is NPPC [18]. Other extension versions of TWSVM also can be contained in the optimization problem (17), for instance, smooth TWSVM, 1-norm LS-TWSVM [17], and so forth, in which we just need to select proper norm or loss function.

More importantly, our framework can solve multiclass classification problem, which is extension of TWSVM, TBSVM, LS-TWSVM, NPPC, and so forth. It should be pointed out that our framework is not straight extension of TWSVM and its deformation versions. Concretely, from the optimization problem (17), we can see that the first term contains the patterns except for those of the *k*th class and the third term just involves the patterns of the *k*th class. This strategy cannot lead to significant increase of the complexity of the optimization when the number *K* of classes increases. We will dwell on in specific algorithm afterwards.

Now, we give the detailed algorithm to the hinge loss function as an example, called hinge NPSVM (HNPSVM). And then the optimization problem (17) is the following formulation with the hinge loss function:

(19) minwk,bk12Bkwk+ek2bk22+12Ck∗wk22+bk2 +Ckek1Tmax0,ek1−Akwk+ek1bk,

where the matrix *A*_*k*_ is comprised of the patterns in the *k*th class, the matrix *B*_*k*_ is defined (12), *C*_*k*_^*∗*^ ≥ 0 and *C*_*k*_ > 0 are the parameters, *e*_*k*1_ and *e*_*k*2_ are vectors of ones of appropriate dimensions, and *k* = 1,2,…, *K*. Actually, the problem is equivalent to the following quadratic programming:

(20) minwk,bk,ξk Bkwk+ek2bk22+12Ck∗wk22+bk2+Ckek1Tξk,s.t. hiiAkwk+ek1bk+ξk≥ek1, ξk≥0,

where the matrix *A*_*k*_ is comprised of the patterns in the *k*th class, the matrix *B*_*k*_ is defined (12), *C*_*k*_^*∗*^ ≥ 0 and *C*_*k*_ > 0 are the parameters, *e*_*k*1_ and *e*_*k*2_ are vectors of ones of appropriate dimensions, and *k* = 1,2,…, *K*.

In fact, for *k* = 1,2,…, *K*, we have *K* QP problems like (20). In particular, when *K* is equal to 2, that is, *k* = 1,2, the QP problems (4) and (5) can be obtained as a special case of (20) with *C*_*k*_^*∗*^ = 0. For simplicity, assume that the number of each class points is almost balanced; namely, the number of the *k*th class is *l*_*k*_ = *l*/*K*. Then, note that the constraints just involve the patterns of the *k*th class, so the complexity of the the problem (20) is no more than (*l*_*k*_)^3^ = (*l*/*K*)^3^. However, if TWSVM is directly extended to multiclass classification case like [22], we will get a different optimization problem, in which the roles of patterns of the *k*th class and the rest class are switched. Thus, the complexity of the optimization problem will increase significantly and is determined by the patterns except for the patterns of the *k*th class in the training set (10), which is no more than ((*K* − 1)(*l*/*K*))^3^. Obviously, our approach is approximately (*K* − 1)^3^ times faster than the model in [22]. On the other hand, when the number of each class points is unbalanced, our apprach still is faster than the model in [22] because the complexity of our optimization problem just is decided by the number of the patterns of the *k*th class rather than the patterns of the rest classes. Therefore, our HNPSVM keeps the computation complexity low.

It is well known that the solution of primal problem (20) is obtained from the solutions of their dual problems. So we now derive their dual problems. The Lagrangian function of the problem (20) is given by

(21) Lwk,bk,ξk,αk,ηk =12Bkwk+ek2bk22+12Ck∗wk22+bk2+Ckek1ξk  −αkTAkwk+ek1bk+ξk−ek1−ηkTξk,

where *α*_*k*_, *η*_*k*_ are nonnegative Lagrange multiplier vectors. The Karush-Kuhn-Tucker (KKT) necessary and sufficient optimality conditions [26] for the QP problem (20) are given by

(22) $$\begin{array}{c} \nabla_{w_{k}}L=B_{k}^{T}\left(B_{k}w_{k}+e_{k2}b_{k}\right)+C_{k}^{*}w_{k}-A_{k}^{T}\alpha_{k}=0, \end{array}$$

(23) $$\begin{array}{c} \nabla_{b_{k}}L=e_{k2}^{T}\left(B_{k}w_{k}+e_{k2}b_{k}\right)+C_{k}^{*}b_{k}-e_{k1}^{T}\alpha_{k}=0, \end{array}$$

(24) $$\begin{array}{c} \nabla_{\xi_{k}}L=C_{k}e_{k1}-\alpha_{k}-\eta_{k}=0, \end{array}$$

(25) $$\begin{array}{c} \left(A_{k}w_{k}+e_{k1}b_{k}\right)+\xi_{k}\geq e_{k1},\;\xi_{k}\geq 0, \end{array}$$

(26) $$\begin{array}{c} -\alpha_{k}^{T}\left(\left(A_{k}w_{k}+e_{k1}b_{k}\right)+\xi_{k}-e_{k1}\right)=0,\;\;\eta_{k}^{T}\xi_{k}=0, \end{array}$$

(27) $$\begin{array}{c} \alpha_{k}\geq 0,\;\;\eta_{k}\geq 0. \end{array}$$

Since *η*_*k*_ ≥ 0, according to (24), we have

(28) $$\begin{array}{c} 0\leq \alpha_{k}\leq C_{k}. \end{array}$$

Next, from (22) and (23), we can obtain

(29) $$\begin{array}{c} \left(\left[\begin{array}{cc} B_{k}^{T} & e_{k}^{T} \end{array}\right]\left[\begin{array}{cc} B_{K} & e_{k} \end{array}\right]+C_{k}^{*}I\right){\left[\begin{array}{cc} w_{k}^{T} & b_{k} \end{array}\right]}^{T}-\left[\begin{array}{cc} A_{k}^{T} & e_{k}^{T} \end{array}\right]\alpha_{k}=0, \end{array}$$

where *I* is an identity matrix of appropriate dimensions. Let $v_{k}={\left[\begin{array}{cc} w_{k}^{T} & b_{k} \end{array}\right]}^{T}$; (29) can be written as

(30) HkTHk+CkIvk−GkTαk=0,or  vk=HkTHk+CkI−1GkTαk,

where $H_{k}=\left[\begin{array}{cc} B_{k} & e_{k2} \end{array}\right]$ and $G_{k}=\left[\begin{array}{cc} A_{k} & e_{k1} \end{array}\right]$. And then putting (30) into the Lagrangian function (21) and using (22)–(28), we can get the dual problem of the primal problem (20):

(31) maxαk ek1Tαk−12αkTHkGkTGk+Ck∗I−1HkTαk,s.t. h0≤αk≤Ck,

where *C*_*k*_^*∗*^ > 0 and *C*_*k*_ > 0 are parameters and *k* = 1,2,…, *K*. Obviously, if we have the solution of the QP problem (31), then we obtain the *K* nonparallel hyperplanes (16) by  (30).

It is worth mentioning that the parameter *C*_*k*_^*∗*^ replaces *ϵ* as in (8), so *C*_*k*_^*∗*^ is no longer a fixed small scalar but a weighting factor which determines the trade-off between the regularization term and the empirical risk in the problem (20). Therefore, the high and low of the value of *C*_*k*_^*∗*^ reflects the structure of minimization principle and our HNPSVM includes MBSVM.

### 3.2. Nonlinear Framework

Similarly, we also extend the linear framework of NPSVMs to nonlinear case. For a *K*-class classification (10), our goal is to find *K* kernel-generated hyperplanes:

(32) $$\begin{array}{c} K\left(x,A^{T}\right)u_{k}+b_{k}=0,\;\;k=1,\ldots ,K, \end{array}$$

where *A* = [*A*_1_,…, *A*_*k*_] and *K*(*x*, *A* ^T^) is an appropriately chosen kernel function.

In order to obtain the *K* hyperlanes (32), we construct the following framework formulation:

(33) minuk,bk12KBkT,ATuk+ek2bk22+12Ck∗uk22+bk2 +Ckek1TLek1,KAkT,ATuk+ek1bk,

where the matrix *A*_*k*_ is comprised of the patterns in the *k*th class, the matrix *B*_*k*_ is defined (12), *C*_*k*_^*∗*^ ≥ 0 and *C*_*k*_ > 0 are the parameters, *e*_*k*1_ and *e*_*k*2_ are vectors of ones of appropriate dimensions, *k* = 1,2,…, *K*, and *L*(·, ·) is the loss function (e.g., square loss or hinge loss, etc.). Similarly, as discussed in the last subsection, the problem (33) can be reduced to the nonlinear formulations of the difference approaches (e.g., TWSVM, TBSVM, LS-TWSVM, NPPC, etc.) when the difference loss functions or parameters are selected for *K* = 2.

A new pattern *x* ∈ *R* ^*n*^ is assigned to the *k*th class by the following decision functions:

(34) $$\begin{array}{c} f\left(x\right)=arg\underset{k=1,\ldots ,K}{max}\left|K\left(x,A^{T}\right)u_{k}+b_{k}\right|, \end{array}$$

where |·| is the absolute value. Note that, in this decision function (34), we just compute the absolute value rather than Euclidean distance from the pattern *x* to the hyperplanes. This strategy reduces the complexity of computation because Euclidean distance should be $|K(x,A^{T})u_{k}+b_{k}|/\sqrt{u_{k}^{T}K(A^{T},A^{T})u_{k}}$ from the pattern *x* to the *k*th hyperplanes. Thus, the decision function (34) not only saves the computation quantity but also keeps the consistency with the first term of the problem (33).

Now, we still select the hinge loss function as example. Then, the problem (33) can be formulated as follows:

(35) minuk,bk,ξk 12KBkT,ATuk+ek2bk22    +12Ck∗uk22+bk2+Ckek1Tξk,s.t. hhKAkT,ATuk+ek1bk+ξk≥ek1,   ξk≥0,

where the matrix *A*_*k*_ is comprised of the patterns in the *k*th class, the matrix *B*_*k*_ is defined by (12), *C*_*k*_^*∗*^ > 0 and *C*_*k*_ > 0 are parameters, *e*_*k*1_ and *e*_*k*2_ are vectors of ones of appropriate dimensions, and *k* = 1,2,…, *K*. Similarly, derived process with the linear case, its dual problem is formulated as:

(36) maxαk ek1Tαk−12αkTRkSkTSk+Ck∗IRkTαk,s.t. h0≤αk≤Ck,

where *C*_*k*_^*∗*^ > 0 and *C*_*k*_ > 0 are parameters, $R_{k}=\left[\begin{array}{cc} K(A_{k}^{T},A^{T}) & e_{k1} \end{array}\right]$, $S_{k}=\left[\begin{array}{cc} K(B_{k}^{T},A^{T}) & e_{k2} \end{array}\right]$, and *k* = 1,2,…, *K*. And the augmented vector $z_{k}={\left[\begin{array}{cc} u_{k} & b_{k} \end{array}\right]}^{T}$ is given by *z*_*k*_ = (*S*_*k*_^T^ *S*_*k*_ + *C*_*k*_^*∗*^ *I*)^−1^ *R*_*k*_^T^ *α*_*k*_.

### 3.3. SOR Algorithm

In our HNPSVMs, the QP problems (31) and (36) can be rewritten as the following unified forms:

(37) minα   12αTQα−eTα,s.t.   h0≤α≤C,

where *Q* ∈ *R* ^*m*×*m*^ is positive definite. For example, the above problem becomes the problem (36), when *Q* = *R*_*k*_(*S*_*k*_^T^ *S*_*k*_ + *C*_*k*_^*∗*^ *I*)^−1^ *R*_*k*_^T^, *C* = *C*_*k*_.

The above problem (37) can be solved efficiently by the following successive overrelaxation (SOR) algorithm; see [27].

**Algorithm 1 .**

SOR for the QP problem (36) is as follows.

(1) Select the parameter *t*_*k*_ ∈ (0,2) and the initial value *α*_*k*_^0^ ∈ *R* ^*m*_*k*_^.
(2) Suppose that *α*_*k*_^*r*^ is obtained by the *r* times iterate; compute *α*_*k*_^*r*+1^ according to the following iterate formula:
  (38) $$\begin{array}{c} \alpha_{k}^{r+1}={\left(\alpha_{k}^{r}-t_{k}D_{k}^{-1}\left(Q_{k}\alpha_{k}^{r}-e_{k2}+L_{k}\left(\alpha_{k}^{r+1}-\alpha_{k}^{r}\right)\right)\right)}_{♯}, \end{array}$$
  where *Q* = *R*_*k*_(*S*_*k*_^T^ *S*_*k*_ + *C*_*k*_^*∗*^ *I*)^−1^ *R*_*k*_^T^. And define *L*_*k*_ + *D*_*k*_ + *L*_*k*_^T^ = *Q*_*k*_, where *L*_*k*_ ∈ *R* ^*m*_*k*_×*m*_*k*_^ and *D*_*k*_ ∈ *R* ^*m*_*k*_×*m*_*k*_^ are the strictly lower triangular matrix and the diagonal matrix, respectively.
(3) Stop if ||*α*_*k*_^*r*+1^ − *α*_*k*_^*r*^|| is less than some desired tolerance. Else, replace *α*_*k*_^*r*^ by *α*_*k*_^*r*+1^ and *r* by *r* + 1 and go to 2.

SOR is an excellent TWSVM solver, because it can process efficiently very large datasets that need not reside in memory. Furthermore, it has been proved that this algorithm converges linearly to a solution in [27, 28]. It should be pointed out that we employ the Sherman-Morrison-Woodbury formula [29] for the inversion of matrix (*S*_*k*_^T^ *S*_*k*_ + *C*_*k*_^*∗*^ *I*) and, hence, need only to invert matrix with a lower order *l*_*k*_, instead of the order *l*. Further, in practise, if the number of patterns in the *k*th classe is large, then the rectangular kernel technique [30, 31] can be applied to reduce the dimensionality of our nonlinear classifiers.

### 3.4. Several Others Approaches

In this section, we briefly give several extension versions based on our framework by selecting different loss function or replacing 2-norm.

First, if the square loss function is chosen, that is, *L*(1, *g*(*x*)) = (1 − *g*(*x*))^2^, then we can get the following formulation from the framework (17):

(39) minwk,bk 12Bkwk+ek2bk22+12Ck∗wk22+bk2+CkξkTξk,s.t. hAkwk+ek1bk+ξk=ek1,

where the matrix *A*_*k*_ is comprised of the patterns in the *k*th class and the matrix *B*_*k*_ is defined by (12), *C*_*k*_^*∗*^ > 0 and *C*_*k*_ > 0 are parameters, *e*_*k*1_ and *e*_*k*2_ are vectors of ones of appropriate dimensions, and *k* = 1,2,…, *K*. This is extension version of LS-TWSVM [14].

Second, if we replace 2-norm with 1-norm in the problem (39), then we can get the extension of 1-norm LS-TWSVM [17] as follows:

(40) minwk,bk Bkwk+ek2bk1+Ck∗wk1+bk+Ckξk1,s.t. hAkwk+ek1bk+ξk=ek1,

where the matrix *A*_*k*_ is comprised of the patterns in the *k*th class, the matrix *B*_*k*_ is defined by (12), *C*_*k*_^*∗*^ > 0 and *C*_*k*_ > 0 are parameters, *e*_*k*1_ and *e*_*k*2_ are vectors of ones of appropriate dimensions, and *k* = 1,2,…, *K*.

Third, if the loss function is a convex combination of linear and square loss, that is, *L*(1, *g*(*x*)) = *δ*(1 − *g*(*x*))+(1 − *δ*)(1 − *g*(*x*))^2^, where *δ* ∈ (0,1), then we can obtain extension version of NPPC [18] as follows:

(41) minwk,bk 12Bkwk+ek2bk22+12Ck∗wk22+bk2    +Ckδek1Tξk+1−δξkTξk,s.t. hAkwk+ek1bk+ξk=ek1,

where the matrix *A*_*k*_ is comprised of the patterns in the *k*th class, the matrix *B*_*k*_ is defined by (12), *C*_*k*_^*∗*^ > 0 and *C*_*k*_ > 0 are parameters, *e*_*k*1_ and *e*_*k*2_ are vectors of ones of appropriate dimensions, and *k* = 1,2,…, *K*.

Forth, if the square hinge loss function is selected, that is, *L*(1, *g*(*x*)) = (max(0,1 − *g*(*x*)))^2^, then we can get the extension version of smooth TWSVM as follows:

(42) minwk,bk,ξk Bkwk+ek2bk22+12Ck∗wk22+bk2+CkξkTξk,s.t. hiiAkwk+ek1bk+ξk≥ek1,   ξk≥0,

where the matrix *A*_*k*_ is comprised of the patterns in the *k*th class, the matrix *B*_*k*_ is defined by (12), *C*_*k*_^*∗*^ > 0 and *C*_*k*_ > 0 are parameters, *e*_*k*1_ and *e*_*k*2_ are vectors of ones of appropriate dimensions, and *k* = 1,2,…, *K*.

These approaches have the same decision function (18) and can be extended into nonlinear case. And their solving methods can construct based on their binary algorithms.

## 4. Numerical Experiments

In this section, we present experimental results of our binary HNPSVM (BHNPSVM) and multiclass HNPSVM (MHNPSVM) on both artificial and benchmark datasets. In experiments, we focus on the comparison between our methods and some state-of-the-art classification methods, including SVM, GEPSVM, TWSVM, “1-v-1,” “1-v-r,” and MBSVM. All the classification methods are implemented in MATLAB 7.0 [32] environment on a PC with Intel P4 processor (2.9 GHz) with 1 GB RAM. In order to give the fastest training speed, we employ Libsvm [33] to implement the SVM, “1-v-1,” and “1-v-r”. Our BHNPSVM and MHNPSVM and TWSVM and MBSVM are implemented using SOR technique; GEPSVM is implemented by simple MATLAB functions like “eig,” respectively. As for the problem of selecting parameters, we employ standard 10-fold cross-validation technique [34]. Furthermore, the parameters for all methods are selected from the set {2^−8^,…, 2^8^}.

### 4.1. Toy Examples

**Figure 1:**
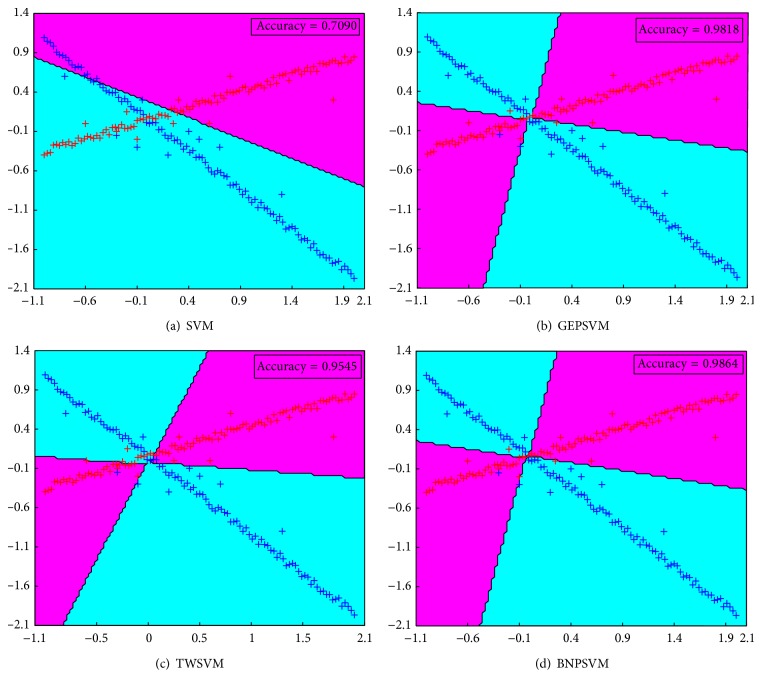
Results of linear SVM, GEPSVM, TWSVM, and BNPSVM on Example 1 dataset.

Firstly, we consider a simple two-dimensional “Cross Planes” dataset as Example 1, which was tested in [11, 13] to indicate that nonparallel hyperplanes classifiers can handle the cross planes dataset much better compared with parallel ones. Now, we show that our BHNPSVM also can handle cross-planes type data well due to use of our decision function. The “Cross Planes” dataset is generated by perturbing points lying on two intersecting lines. Figures 1(a)–1(d) show the dataset and the linear classifiers obtained by SVM, GEPSVM, TWSVM, and our BNPSVM. It is easy to see that the result of our BNPSVM is more reasonable than that of SVM, and better than that of GEPSVM and TWSVM. In addition, we list the accuracy and CPU time for these four classifiers in Table 1. From Table 1, we can see that our BNPSVM obtains the best accuracy while not the slowest computing time.

**Figure 2:**
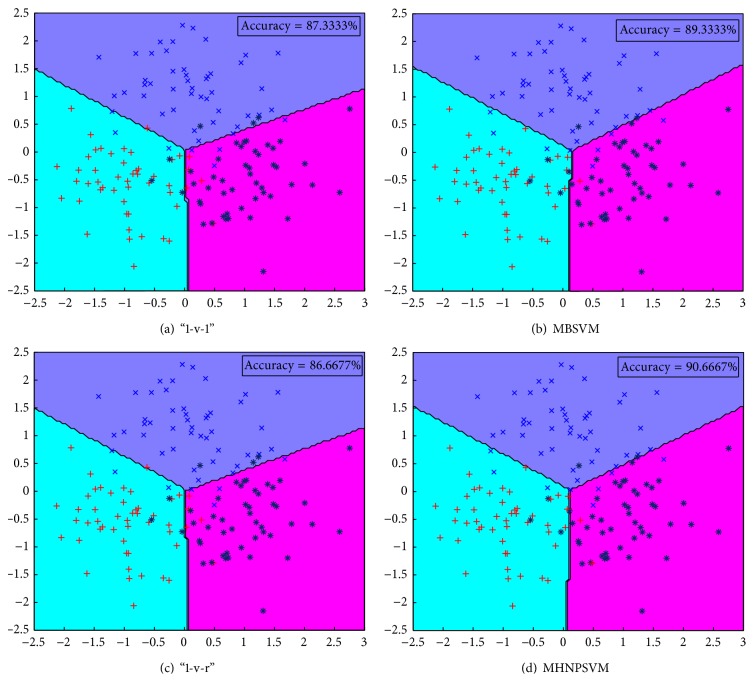
Results of linear “1-v-1,” “1-v-r,” MBSVM, and MHNPSVM on Example 2 dataset.

Secondly, we consider a two-dimensional three-class dataset as Example 2 to show the operating mechanism of our MNPSVM and other multiple-class classifiers. The three-class dataset is generated by perturbing points lying on three intersecting lines. Figures 2(a)–2(d) show the dataset and the linear classifiers obtained by “1-v-1,” “1-v-r,” MBSVM, and MHNPSVM. It is easy to see that the result of MBSVM and MHNPSVM is more reasonable than that of “1-v-1” and “1-v-r.” We also list the accuracy and CPU time of Example 2 for these four classifiers in Table 1. From Table 1, we can see that our MHNPSVM obtains the best accuracy in all these two examples, indicating that our MHNPSVM is suitable for both “Cross Planes” and multiclass problems.

### 4.2. Benchmark Datasets

In order to further compare our methods with others, we examine nine binary-class datasets and nine multiclass datasets used by [12, 35], from the UCI Repository of machine learning database [36].  Table 2 gives the details of these eighteen datasets.

In order to compare the behavior of our linear BHNPSVM with SVM, GEPSVM, and TWSVM, the numerical experimental results for binary-class UCI datasets are summarized in Table 3. In Table 3, the classification accuracy and computation time are listed. In Table 3, the best accuracy is shown by bold figures. It is easy to see that most of the accuracies of our linear BHNPSVM are better than linear SVM, GEPSVM, and TWSVM on these datasets. It can also be seen that our BHNPSVM is a little faster than TWSVM and is competitive with SVM (implements by Libsvm). We also list the mean accuracy and mean time for these four classifiers. Our BHNPSVM gains the the highest mean accuracy while faster training speed than TWSVM.

Table 4 is concerned with our kernel BHNPSVM, SVM, GEPSVM, and TWSVM on binary-class UCI datasets. The Gaussian kernel *K*(*x*, *x*′) = *e* ^−*μ*||*x* − *x*′||^2^^ is used. The kernel parameter *μ* is also obtained through searching from the range from 2^−8^ to 2^8^. The training CPU times for these four classifiers are also listed. The results in Table 4 are similar to those appearing in Table 3 and therefore confirm the above conclusion further.

In order to compare the behavior of our MHNPSVM with other multiple-class classifiers, we compare our MHNPSVM with “1-v-1,” “1-v-r,” and MBSVM, the linear results of numerical experiments on multiclass UCI datasets are summarized in Table 5. In Table 5, the classification accuracy and computation time are listed.

From Table 5, we can see that the accuracy of linear MHNPSVM is significantly better than linear MBSVM on all 9 UCI datasets. We also obtain that MHNPSVM and MBSVM are almost same fast because they both solve two SOR algorithms instead of two QP problems with the same size. In contrast, classification accuracy of “1-v-1” and “1-v-r” is no statistical difference with MHNPSVM for all cases except for vowel dataset, and “1-v-1” and “1-v-r” are a bit lower than MHNPSVM and MBSVM in average training time. Thus, with the proposed formulation of MHNPSVM allows the classifier to learn better by reducing the generalization errors. However, this improved performance is obtained at the cost of more tuning effort involved. This is because MHNPSVM requires tuning of more parameters than MBSVM.

Table 6 shows the nonlinear MHNPSVM with “1-v-1,” “1-v-r,” and MBSVM, the results of numerical experiments. In Table 6, the classification accuracy and computation time are listed. The results in Table 6 are similar to those appearing in Table 5; MHNPSVM has better classification accuracy than MBSVM in eight datasets, while MBSVM is better than MHNPSVM in one dataset, and MHNPSVM and MBSVM are much faster than “1-v-1” and “1-v-r”, especially when the amount of data increases.

## 5. Conclusions

In this paper, a general framework of nonparallel hyperplanes support vector machines, termed NPSVMs, are proposed for binary classification and multiclass classification. For binary classification, this framework includes TWSVM and its many deformation versions, for instance, TWSVM, TBSVM, LS-TWSVM, NPPC, and so forth, when different loss functions and parameters are selected. For multiclass classification, we do not directly extend TWSVM and its deformation versions to get the framework, in which we switch the roles of the patterns of the *k*th class and the rest classes. This strategy does not lead to significant increase of the computation complexity when the number of classes is increasing. Moreover, in the decision function, “min” and Euclidean distance in TWSVM are replaced by “max” and the absolute value |*w* ^T^ *x* + *b*|, respectively. The absolute value |*w* ^T^ *x* + *b*| is not only simpler but also more consistent with the primal problems. In particular, we discuss the linear and nonlinear case of the framework with the hinge loss function as example. Moreover, we also give the primal problems of several extensions of TWSVM's deformation versions. The numerical experiments on several artificial and benchmark datasets indicate that our NPSVMs yield comparable generalization performance compared with SVM, GEPSVM, TWSVM, MBSVM, “1-v-1,” and “1-v-r”. In short, the proposed framework not only includes TWSVM and its many deformation versions but also extends them into multiclass classification under keeping the merit of TWSVM (learning speed).

In the future, we will develop the idea of nonparallel hyperplanes classifiers to other problems such as ordinal regression, multi-instance, and multilabel classification.

## Figures and Tables

**Table 1:** Tenfold testing percentage test set accuracy (%) on example data sets.

Data set	SVM	GEPSVM	TWSVM	BHNPSVM
	Accuracy %	Accuracy %	Accuracy %	Accuracy %
	Time (s)	Time (s)	Time (s)	Time (s)
Example 1	70.90	95.45	98.18	**98.64**
(202 × 2)	0.122	0.0005	0.0064	0.0052

Data set	“1-v-1”	“1-v-r”	MBSVM	MHNPSVM
	Accuracy %	Accuracy %	Accuracy %	Accuracy %
	Time (s)	Time (s)	Time (s)	Time (s)

Example 2	87.33	86.67	89.33	**90.67**
(330 × 2)	0.098	0.0006	0.0079	0.0095

**Table 2:** The detailed characteristics of the datasets.

Data	#Ins	#Fea	#class	Data	#Ins	#Fea	#class
Hepatitis	155	19	2	Votes	435	16	2
WBPC	198	34	2	Sonar	208	60	2
Heart-statlog	270	13	2	BUPA	345	6	2
Pima-Indian	768	8	2	CMC	1473	9	2
Australian	690	14	2	Iris	150	3	4
Wine	178	3	13	Ecoli	336	8	8
Vowel	528	11	10	Glass	214	6	13
Vehicle	846	4	18	Car	1728	6	4
Segment	2310	7	19	Satimage	4435	6	36

#Ins is the number of the training points; #attributes is the number of attributes; #class is the number of class.

**Table 3:** Tenfold testing percentage test set accuracy (%) on binary-class UCI data sets for linear classifiers.

Data sets	TWSVM	SVM	GEPSVM	BHNPSVM
	Accuracy %	Accuracy %	Accuracy %	Accuracy %
	Time (s)	Time (s)	Time (s)	Time (s)
Hepatitis	82.89 ± 6.30^*^	84.13 ± 5.58	80.07 ± 5.43	**85.47 ± 1.36** ^*^
	0.012	0.012	0.0006	0.0304
BUPA liver	66.40 ± 7.74^*^	67.78 ± 5.51	61.33 ± 6.26	**69.97 ± 0.56** ^*^
	0.840	0.0549	0.0012	0.2143
Heart-statlog	**84.44 ± 6.80**	83.12 ± 5.41	75.37 ± 7.02	**84.44 ± 0.56**
	0.023	0.0281	0.0022	0.1092
Votes	95.85 ± 2.75	95.80 ± 2.65	91.93 ± 3.18	**95.58 ± 2.75**
	0.797	1.1446	0.0039	0.1027
WPBC	**83.68 ± 5.73** ^*^	83.30 ± 4.53	76.76 ± 6.67	81.32 ± 1.36^*^
	0.012	0.0432	0.0002	0.0465
Sonar	77.00 ± 6.10	**80.13 ± 5.43**	73.16 ± 8.33	74.15 ± 1.73
	0.007	0.0946	0.0225	0.007
Australian	85.94 ± 5.84	**88.51 ± 4.85**	80.00 ± 3.99	85.27 ± 3.26
	0.3460	0.2350	0.0029	0.4250
Pima-Indian	73.80 ± 4.97^*^	**77.34 ± 4.37**	75.47 ± 4.64	77.05 ± 0.48^*^
	0.121	0.261	0.0016	0.4793
CMC	68.28 ± 2.21^*^	67.82 ± 2.63	66.76 ± 2.98	**77.86 ± 0.22** ^*^
	1.247	0.597	0.0050	1.197

Mean accuracy	79.81	80.88	75.65	**81.23**
Mean time	0.38	0.27	0.004	0.29

^*∗*^A greater difference between BHNPSVM and TWSVM.

**Table 4:** Tenfold testing percentage test set accuracy (%) on binary-class UCI datasets for nonlinear classifiers.

Datasets	TWSVM	SVM	GEPSVM	BHNPSVM
	Accuracy %	Accuracy %	Accuracy %	Accuracy %
	Time (s)	Time (s)	Time (s)	Time (s)
Hepatitis	83.39 ± 7.31	**84.13 ± 6.25**	80.00 ± 5.2	83.40 ± 3.58
	0.016	0.0142	0.0035	0.0697
BUPA liver	67.83 ± 6.49^*^	68.32 ± 7.20	63.01 ± 7.46	**74.24 ± 0.64** ^*^
	0.033	0.0129	1.305	0.1522
Heart-statlog	82.96 ± 4.67^*^	83.33 ± 9.11	**86.52 ± 7.36**	84.04 ± 4.56^*^
	0.029	0.0250	0.438	0.1120
Votes	94.91 ± 4.37	**95.64 ± 7.23**	94.5 ± 3.37	95.21 ± 5.18
	0.072	0.0495	0.087	0.0152
WPBC	**81.28 ± 5.92**	80.18 ± 6.90	80.07 ± 5.97	80.89 ± 1.17
	0.029	0.0148	0.0043	0.0468
Sonar	**89.64 ± 6.11**	88.93 ± 10.43	81.93 ± 4.41	88.05 ± 1.79
	0.014	0.0781	0.020	0.2896
Australian	75.8 ± 4.91^*^	**85.51 ± 4.85**	69.55 ± 5.37	77.58 ± 2.53^*^
	0.420	0.0425	0.334	0.497
Pima-Indian	73.74 ± 5.2^*^	76.09 ± 3.58	74.66 ± 5.00	**77.70 ± 0.39** ^*^
	0.427	0.442	15.892	0.381
CMC	73.95 ± 3.48^*^	68.98 ± 3.44	68.67 ± 3.84	**78.43 ± 0.13** ^*^
	1.708	1.755	1.042	1.920

Mean accuracy	80.39	81.23	77.66	**82.17**
Mean time	0.3053	0.27	2.1251	0.3871

^*∗*^A greater difference between BHNPSVM and TWSVM.

**Table 5:** Tenfold testing percentage test set accuracy (%) on multiclass UCI datasets for linear classifiers.

Dataset	1-v-1	1-v-r	MBSVM	MHNPSVM
	Accuracy (%)	Accuracy (%)	Accuracy (%)	Accuracy (%)
	Time (s)	Time (s)	Time (s)	Time (s)
Iris	96.83 ± 1.75	95.73 ± 3.78	95.00 ± 4.95	**96.96 ± 1.12**
	0.025	0.014	0.009	0.010
Wine	96.59 ± 1.48	**97.72 ± 0.74**	94.77 ± 4.07	95.88 ± 2.21
	0.058	0.021	0.028	0.023
Ecoli	**87.63 ± 0.81**	86.77 ± 0.87	85.72 ± 1.02	86.78 ± 0.75
	0.863	0.522	0.097	0.089
Vowel	54.21 ± 2.24	57.44 ± 3.26	59.42 ± 4.96^*^	**64.60 ± 3.06** ^*^
	1.459	0.580	0.160	0.172
Glass	94.16 ± 1.84	94.42 ± 4.06	92.80 ± 9.80^*^	**95.83 ± 1.04** ^*^
	1.037	0.405	0.183	0.105
Vehicle	77.79 ± 2.21	**78.22 ± 2.10**	77.59 ± 2.16	77.13 ± 1.87
	28.11	10.05	2.96	2.58
Car	86.78 ± 0.50	86.72 ± 0.31	84.09 ± 0.33^*^	**87.79 ± 0.91** ^*^
	16.042	13.79	5.92	6.05
Segment	91.60 ± 2.428	**92.54 ± 2.03**	92.68 ± 1.87	93.04 ± 2.01
	28.078	15.26	17.04	17.55
Satimage	91.80 ± 0.81	90.20 ± 1.13	**92.40 ± 2.08**	91.40 ± 1.49
	60.50	32.29	47.45	45.27

Mean accuracy	86.38	86.64	86.05	**87.71**
Mean time	15.13	8.10	8.21	7.98

^*∗*^A greater difference between MHNPSVM and MBSVM.

**Table 6:** Tenfold testing percentage test set accuracy (%) on multiclass UCI datasets for nonlinear classifiers.

Dataset	1-v-1	1-v-r	MBSVM	MHNPSVM
	Accuracy (%)	Accuracy (%)	Accuracy (%)	Accuracy (%)
	Time (s)	Time (s)	Time (s)	Time (s)
Iris	**98.93 ± 1.11**	97.63 ± 5.46	98.12 ± 2.08	98.74 ± 1.92
	0.0054	0.0264	0.037	0.030
Wine	97.08 ± 3.32	**97.72 ± 0.86**	96.45 ± 1.29	97.28 ± 0.96
	7.294	4.6504	0.592	0.523
Ecoli	92.27 ± 1.03	90.35 ± 0.47	91.06 ± 1.45^*^	**92.95 ± 0.89** ^*^
	0.382	0.0843	0.154	0.182
Glass	98.09 ± 1.04	99.14 ± 0.97	98.76 ± 1.22	**99.24 ± 0.93**
	0.692	0.1085	0.089	0.092
Vowel	91.37 ± 0.86	**94.32 ± 0.18**	80.42 ± 4.37^*^	85.86 ± 4.72^*^
	1.482	0.3844	0.623	0.593
Vehicle	81.03 ± 5.73	82.49 ± 4.26	82.01 ± 1.33	**83.57 ± 1.79**
	19.562	11.456	2.81	2.50
Car	**88.37 ± 0.55**	87.36 ± 0.68	85.74 ± 0.33	86.57 ± 0.46
	3.6571	0.9405	1.832	1.944
Segment	95.15 ± 6.02	94.65 ± 4.38	**95.96 ± 4.08**	95.90 ± 3.29
	128.42	91.69	53.27	49.58
Satimage	93.80 ± 1.46	93.05 ± 1.46	94.03 ± 1.93	**94.47 ± 1.58 **
	190.27	132.47	89.05	88.36

Mean accuracy	92.90	**92.97**	91.39	92.73
Mean time	39.08	26.87	16.50	15.98

^*∗*^A greater difference between MHNPSVM and MBSVM.
